# Iceman Survived due to Cooling Device

**DOI:** 10.5402/2011/617912

**Published:** 2011-05-09

**Authors:** M. Roser, F. Martens, C. Storm

**Affiliations:** ^1^Department of Internal Medicine/Cardiology, German Heart Institute Berlin, Augustenburger Platz 1, 13353 Berlin, Germany; ^2^Department of Nephrology and Intensive Care Medicine, Charité Universitaetsmedizin Berlin, Augustenburger Platz 1, 13353 Berlin, Germany

## Abstract

Although mild hypothermia treatment is part of the standard postresuscitation care today, no standard method for treatment of accidental severe hypothermia has been yet established. Different strategies including invasive and noninvasive methods have been described in the literature. We present the case of a 75-year-old man with accidental severe hypothermia (23°C) and demonstrate that using a surface cooling device with automatic controlled temperature feedback mechanism (ArcticSun2000 Medivance, Louisville, Colorado, USA) is an effective and safe method for controlled rewarming in this life-threatening setting.

Mild hypothermia (32–34°C) treatment is recommended in cardiac arrest patients and is now part of standard postresuscitation care [1]. In contrast, no standard method has been established for treatment of accidental severe hypothermia (<28°C). A 75-year-old homeless man was admitted to ICU with an oesophageal temperature of 23°C. He was found on a park bench, and the local weather conditions this October morning were 3.4°C, 91.6% humidity, and 3.9 m/s wind speed. On admission he was conscious with normal reflexes, but not able to answer adequately. The GCS score was 7. He was spontaneously breathing, oxygen saturation was 99% without additional oxygen, ECG showed sinus bradycardia with 30 bpm and blood pressure of 100/50 mmHg (Figure 1). Relevant laboratory findings included Hb 16.4 g/dl, CK 500 U/l, potassium 3.0 mmol/l, pH 7.18, BE −8.5, lactate 10 mg/dl, ethanol <0.1 g/l. Due to stable cardiocirculatory conditions, we were reluctant to apply intubation and ventilation. Thus, a commercially available hypothermia device (ArcticSun2000 Medivance, Louisville, Colorado, USA) with an automatic controlled temperature feedback mechanism was used in the rewarming modus. Initially a quick rewarming phase over 180 minutes from 23°C to 32°C was followed by a maintenance phase of 600 minutes at 33°C and thereafter a stepwise increase of 0.1°C per hour. In addition, only i.v. fluids were given, as there was no need for vasopressors. With increasing body temperature, extracellular potassium level increased into the normal range. During rewarming, neither severe arrhythmias nor any shivering occurred. Within 24 hours, the patient completely recovered and ordered lunch. Clinical examination revealed no pathological findings, especially no neurological deficit. In the follow-up visit three months later, the patient still had Cerebral Performance Category (CPC) of 1. This is a rare case of survived accidental severe hypothermia (23°C) without consequences and demonstrates that aggressive rewarming is also possible in conscious patients. Although a number of methods have been described, the best technique of rewarming in this patient population has not yet been established. Current strategies include invasive (peritoneal lavage, intravasal catheter devices, or cardiocirculatory bypass) and noninvasive (warming blankets, warm fluids, or surface cooling devices) methods [2–5]. The choice of the appropriate method depends on the neurological and circulatory conditions, comorbidities as well as the clinical surrounding and experience of the medical centre. We could demonstrate that using a surface cooling system is an effective and safe method for controlled rewarming in this life-threatening setting. Our patient survived without neurological impairment accidental hypothermia of 23°celsius. 

## Figures and Tables

**Figure 1:**
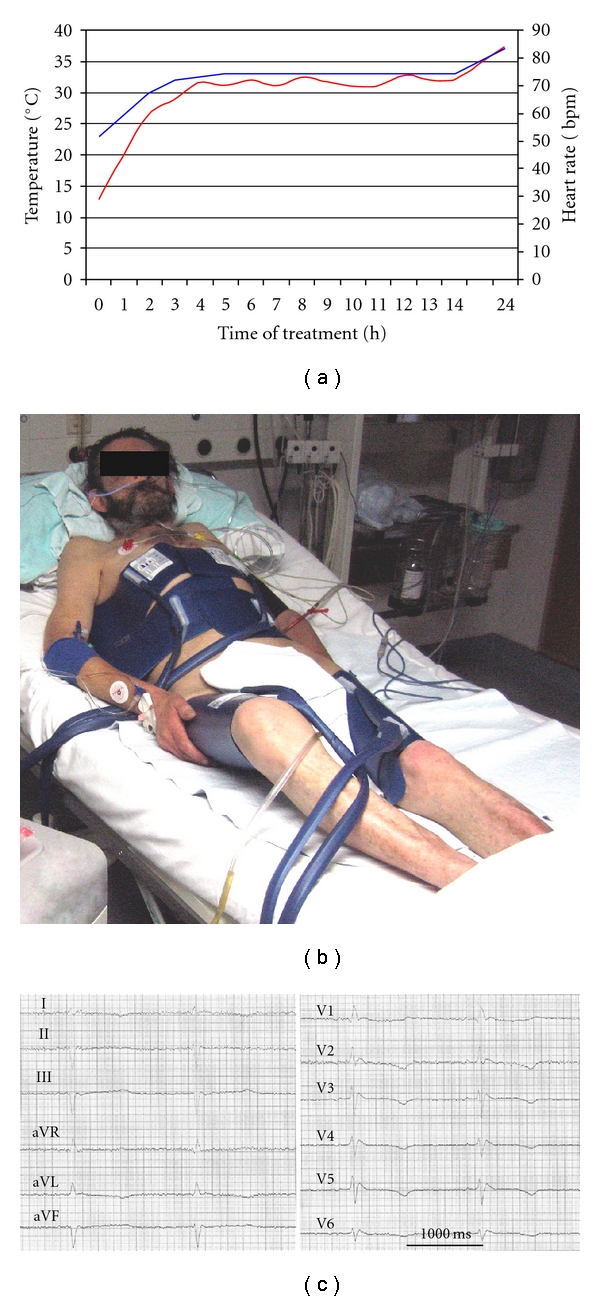
(a) Time course of oesophageal temperature (blue line) and heart rate (red line) over the first 24 hours of treatment. (b) Conscious male patient with 23°C central body temperature on admission at the beginning of rewarming procedure with a surface cooling device. (c) Electrocardiogram 1 hour after hospital admission (body temperature at this time approx. 26°celsius) showing sinus bradycardia with 36 beats per minute (RR interval 1650 ms).
